# Associations of cord leptin and cord insulin with adiposity and blood pressure in White British and Pakistani children aged 4/5 years

**DOI:** 10.12688/wellcomeopenres.15433.1

**Published:** 2019-10-15

**Authors:** Jane West, Gillian Santorelli, Paul Collings, Daniel Bingham, Peter Whincup, Naveed Sattar, Tom Norris, John Wright, Debbie A. Lawlor

**Affiliations:** 1 Bradford Institute for Health Research, Bradford, BD9 6RJ, UK; 2 Population Health Sciences, Bristol Medical School, University of Bristol, Bristol, BS8 2BN, UK; 3 Population Health Research Institute, St George's, University of London, London, SW17 0RE, UK; 4 Institute of Cardiovascular and Medical Sciences, University of Glasgow, Glasgow, G12 8TA, UK; 5 School of Sport, Exercise and Health Sciences, Loughborough University, Loughborough, LE11 3TU, UK

**Keywords:** Cord blood leptin, cord blood insulin, adiposity, ethnicity

## Abstract

**Background:** Cord leptin and cord insulin concentrations may be important biomarkers of child adiposity and cardiovascular health, especially in populations with an increased long-term risk of type 2 diabetes and cardiovascular diseases. We aimed to determine whether cord leptin and insulin are associated with adiposity and early cardiovascular health at age 4/5, and whether any associations differ between White British and Pakistani children.

**Methods:** Using bi-ethnic cohort data from 6060 mother-offspring pairs (2717 (44.8%) White British, 3343 (55.2%) Pakistani), we examined associations of cord leptin and insulin with adiposity (BMI, skinfold thickness) and systolic and diastolic blood pressure at age 4/5.

**Results:** Cord leptin and insulin were higher in Pakistani compared to White British children (7.4 ng/ml versus 6.7 ng/ml and 4.1 mU/L versus 3.63 mU/L **,** respectively). Associations with adiposity measurements were similar in both groups and close to the null value. For example, each 10 ng/ml higher cord leptin was associated with a difference in mean childhood BMI of 0.10 kg/m ^2^ (95% CI 0.01, 0.19) in White British, 0.01 kg/m ^2^ (95% CI -0.08, 0.10) in Pakistani and 0.04 kg/m ^2^ (95% CI -0.02, 0.11) in both groups combined.  Associations with systolic and diastolic blood pressure were also close to the null and consistent in both groups.

**Conclusions: **We found no evidence that cord leptin or insulin were likely to be valuable biomarkers for predicting later adiposity and blood pressure in White British or Pakistani children. For now, other factors such as family history and social-economic status may be more useful markers of risk.

## Introduction

UK South Asian adults have greater total and central adiposity, are more likely to be insulin resistant and have higher risk of type 2 diabetes (T2D) and cardiovascular disease (CVD) than White Europeans ^1– 4^. We, using the same cohort as that used here, have previously found evidence that this greater adiposity may be present at birth, both in Pakistani infants whose parents were born in the UK, as well as those whose parents were South Asian born ^5, 6^. By age 4/5 years, we observed that UK Pakistani children were taller, had lower BMI, lower triceps skinfolds (TSF) and similar subscapular skinfolds (SSF), similar systolic blood pressure (SBP), but higher diastolic blood pressure (DBP) compared with White British children ^7, 8^.

Whilst higher maternal BMI and gestational fasting glucose are positively causally related to birthweight and ponderal index ^9^, we and others have found no strong evidence for an association of maternal gestational diabetes or maternal glucose (fasting or post-load) with offspring BMI, TSF, SSF, SBP or DBP in early or mid-childhood ^8, 10– 12^. We have, however, found that greater maternal pre-pregnancy BMI is associated with subsequent offspring greater adiposity at age 4/5 ^8^. Cord-blood insulin reflects fetal response to maternal gestational glucose levels, and we have shown that it mediates much of the ethnic difference in infant fat mass, as proxied by cord-blood leptin ^13^, a reliable marker of fat mass at birth ^14^. Whist gestational diabetes and glucose may not be associated with adiposity at age 4/5, it is possible that cord leptin and insulin, reflecting infant fat mass and fetal response to glucose, respectively, relate to subsequent child adiposity and cardiovascular health. If they do, then these could potentially be valuable biomarkers of future cardiometabolic disease risk. Several studies have explored the relation of cord leptin with subsequent adiposity but have reported conflicting findings ^15– 17^. Most studies have been small (<600 participants) and have reported positive ^15, 17^ and negative ^16^ associations with childhood BMI. By far the largest study to date (n=2138) examined associations of cord-blood leptin and adiponectin (thought to be positively related to fat mass in early life) with offspring adiposity (total fat mass, BMI and waist circumference) ^18^. It identified a weak positive association between cord leptin and adiposity measures in childhood (age 9–11 years) but not in adolescence (age 15–17 years), with contrasting associations for cord adiponectin (i.e. no strong evidence of association with childhood adiposity but weak positive associations with adolescent adiposity). However, that study was in a largely White European population and to our knowledge, differences in the association between cord leptin and insulin and offspring adiposity or cardiovascular health between South Asian and White European children have not previously been examined. Given the ethnic differences in birth size and cord leptin and insulin, as well as the increased cardiovascular risk seen in South Asian adults, such an investigation could add to our understanding of the extent to which early life biomarkers can identify differing levels of risk between South Asians and White Europeans.

Here, we explore the associations of cord-blood leptin and insulin with offspring adiposity (assessed with BMI, SSF, TSF) and early cardiovascular health (assessed by SBP and DBP) at age 4/5, and examine whether any associations differ between White British and Pakistani origin children born and living in the same UK city. For comparison, we also examine associations of birth weight with adiposity and blood pressure in the two ethnic groups to see if there is any evidence that cord leptin or insulin would be any better at predicting future risk of adverse cardiometabolic health than the more simple and readily available birth weight.

## Methods

### Study population

The Born in Bradford (BiB) cohort study is a prospective pregnancy and birth cohort based in the northern city of Bradford, UK. Full details of the study methodology have been previously reported ^19^. Briefly, to be eligible for the study women had to be booked to give birth in Bradford between March 2007 and December 2010. All pregnant women in Bradford are routinely offered an oral glucose tolerance test at 26–28 weeks gestation and most women were recruited at this appointment where they also gave informed written consent to an interviewer-completed questionnaire ( available on the Born in Bradford website) and had their height and weight recorded. A total of 13,858 children were recruited to the study. There were 172 child deaths and of the remaining 13,686, 11,819 were eligible to start primary school in the school years 2012/13; 2013/14; 2014/15. Of these, 6,947 had a cord blood sample taken at birth. All children in the UK at age 4/5 (reception year) have their height and weight measured by school nurse teams as part of the National Child Measurement Programme (NCMP). For this study, school nurse teams also collected TSF and SSF and SBP and DBP from participating children. Parental opt-out consent is used for the NCMP and we adopted the same approach for the additional skinfold and blood pressure measurements. Ethnic groups other than Pakistani and White British were excluded from these analyses because they included too few participants within each group for meaningful analyses (n=858). We also excluded twins or triplets (n=137) and those with no baseline questionnaire because of recruitment later than the antenatal OGTT recruitment time (n=1019). We excluded those children for whom consent was declined or who could not be matched to, or were not attending a school within the Bradford district (n=1943). Those with missing data on cord blood measures, outcomes or any covariables were also excluded (n=635). The remaining 2,355 mother-offspring pairs (1,127 White British and 1,228 Pakistani) form the complete case sample for this study (see study flow chart, available as *Extended data* ^20^). Ethics approval for the study was granted by Bradford National Health Service Research Ethics Committee (ref 06/Q1202/48).

### Assessment of ethnicity

Ethnicity was self-reported by mothers at the recruitment interview and based on UK Office of National Statistics guidance details of which have been previously reported ^13^.

### Cord blood assays

Cord blood samples were obtained at delivery by the attending midwife. Samples were refrigerated at 4°C in EDTA tubes until collected by laboratory staff within 12 h. Samples were then spun, frozen and stored at −80°C. They were transferred to the Biochemistry Department of Glasgow Royal Infirmary for analysis, where leptin was measured by a highly sensitive in-house ELISA with better sensitivity at lower levels than commercial assays.

### Maternal characteristics and pregnancy data

Height was measured (unshod) at recruitment (26–28 weeks gestation) using a Leicester Height Measure. Weight at first antenatal clinic assessment when women were median 12 weeks (IQR 11, 14) was abstracted from the antenatal records and was used with height measured at recruitment to calculate early pregnancy BMI. Smoking in pregnancy (defined as smoking at any time during pregnancy) was obtained from the recruitment interview. OGTT plasma glucose levels (fasting and 2-hour post-load) were assayed immediately after sampling at the biochemistry department of Bradford Royal Infirmary using the glucose oxidase method on the Siemens Advia 2400 chemistry autoanalysers and the Siemens Advia Centaur assay. Gestational diabetes (GDM) was defined according to modified WHO criteria operating at the time these women were pregnant as either fasting glucose ≥6.1 mmol/l or 2 h glucose ≥7.8 mmol/l ^21^. Women were classified as having gestational hypertension if they had a systolic measure ≥140 and a diastolic ≥90 mmHg on 2 or more occasions after 20 weeks gestation and pre-eclampsia if significant proteinuria (>1+) accompanied hypertension; information on this was obtained from the antenatal records. Parity, gestational age, mode of delivery, birthweight and infant sex were all extracted from clinical records.

### Offspring adiposity measures

Height was measured by trained school nurses using the Leicester Height Measure (Seca) and weight using Seca digital scales, with children unshod and in light clothing. Where height and/or weight were not obtained in school, measures were extracted from primary care datasets, using those recorded closest in time to the skinfold thickness measurements. SSF and TSF measurements were undertaken by the school nurses following training and reliability assessments ^22^. Holtain Tanner/Whitehouse Calipers (Holtain, Crymych, UK) were used and all measurements were taken from the left side of the body whilst the child was seated and had their left arm removed from clothing. All skinfold measurements were conducted within 16 weeks of the NCMP height and weight measures (mean ± SD time difference 13.5 ± 8.0 weeks). High levels of between- and within-school nurse reliability were found for all skinfold measurements ^22^. SBP and DBP measurements were recorded using Omron HEM-907 electronic monitors and were collected at the same time as skinfold thickness measurements and according to a written protocol. The appropriate cuff size (either child or small adult) was used. Children were seated for 2 minutes prior to the BP measurement and all measures were recorded using the left arm. We recorded one BP measurement per child which is consistent with other studies undertaken within a school setting ^23, 24^.

### Statistical analyses

All analyses were undertaken using Stata/SE version 15.1 software. Characteristics of White British and Pakistani origin infants are presented using numbers (%) for categorical variables and mean (SD) or median (IQR) for continuously measured variables. Multivariable linear regression was used to examine the associations of cord blood leptin, insulin and birthweight with offspring BMI, SSF and TSF thickness and SBP and DBP at age 4/5 years in the whole cohort and stratified by the two ethnic groups. Differences in the magnitude or direction of associations were explored by looking at the ethnic specific point estimates and including an interaction term between ethnicity and the exposures for each association. Potential confounders were decided *a priori* based on existing published literature and our previous BiB analyses. In model 1, we adjusted for offspring sex and age (in months) at child measurement. In model 2, we additionally adjusted for potential confounding by maternal age, parity, smoking in pregnancy, BMI, education, housing tenure and whether anyone in the household was in receipt of means-tested benefits and for confounding by adverse pregnancy/perinatal outcomes; gestational age, mode of delivery (lower segment caesarean section [LSCS] or normal), hypertensive disorders of pregnancy (HDP) and gestational diabetes (GDM). Cord-blood levels of leptin and insulin were positively skewed and are described using medians and interquartile range. In regression analyses these were used as exposures (i.e. independent variables) and were included in their original form (ng/ml for leptin and pmol/l for cord insulin) as their skewed distribution did not influence the distribution of model residuals, which were all approximately normal.

## Results

### Maternal and offspring characteristics

Distributions of maternal and offspring characteristics in the sample included in the analyses presented here, are virtually identical to those presented in two recently published papers that included more of the eligible children as they were not restricted to those with cord blood ( Table 1) ^7, 8^. Pakistani mothers were on average slightly older and had a lower BMI than White British mothers. GDM was more common but LSCS less common in Pakistani compared to White British women. Pakistani children were slightly taller, had lower weight, BMI and TSF, similar SSF and SBP, and higher DBP than White British children. Cord leptin and cord insulin levels were higher in Pakistani compared to White British children (7.4 ng/ml versus 6.7 ng/ml and 4.1 mU/L versus 3.63 mU/L, respectively).

**Table 1.** Distributions of maternal and offspring characteristics stratified by ethnicity.

Characteristic	All (n=2355)	White British (n=1127)	Pakistani origin (n=1228)	p-value *
Maternal age at delivery (years) †	27.2 (5.6)	26.9 (6.0)	27.4 (5.1)	0.021
Parity ‖				
0	820 (34.8)	477 (42.3)	343 (27.9)	<0.001
1	751 (31.9)	414 (36.7)	337 (27.4)	
2	438 (18.6)	157 (13.9)	281 (22.9)	
3	213 (9.0)	56 (5.0)	157 (12.8)	
4 or more	133 (5.7)	23 (2.0)	110 (9.0)	
Maternal education ‖				
<5 GCSEs	537 (22.8)	199 (17.6)	338 (27.5)	<0.001
5+ GCSEs	778 (33.0)	409 (36.3)	369 (30.1)	
A Level/equivalent	357 (15.2)	191 (17.0)	166 (13.5)	
Higher than A level	526 (22.3)	220 (19.5)	306 (24.9)	
Other	157 (6.7)	108 (9.6)	49 (4.0)	
Maternal BMI (kg/m ^2^) †	26.2 (5.7)	26.9 (6.0)	25.5 (5.5)	<0.001
Smoked in pregnancy ‖	400 (17.0)	358 (31.8)	42 (3.4)	<0.001
Maternal GDM †	176 (7.5)	55 (4.9)	121 (9.9)	<0.001
Maternal HDP ‖				
Gestational hypertension	167 (7.4)	122 (11.2)	45 (3.8)	<0.001
Pre-eclampsia	54 (2.4)	30 (2.8)	24 (2.0)	
Mode of delivery: LSCS ‖	492 (20.9)	268 (23.8)	224 (18.2)	0.001
Male sex ‖	1150 (48.8)	590 (52.4)	560 (45.6)	0.001
Birthweight (g) †	3278.8 (514.6)	3407.1 (521.7)	3161.1 (478.8)	<0.001
Age at skinfold measurements (years) †	4.6 (0.5)	4.6 (0.5)	4.6 (0.5)	0.453
Child Height (cm) †	108.4 (4.8)	108.2 (4.8)	108.6 (4.9)	0.035
Child Weight (kg) †	18.9 (2.9)	19.1 (2.7)	18.7 (3.0)	0.002
Child BMI (kg/m ^2^) †	16.0 (1.6)	16.3 (1.5)	15.8 (1.7)	<0.001
SSF (mm) †	6.3 (2.2)	6.3 (1.9)	6.3 (2.3)	0.809
TSF (mm) †	10.1 (3.3)	10.7 (3.2)	9.6 (3.3)	<0.001
Cord leptin (ng/ml) ‡	7.1 (3.8-12.8)	6.7 (3.52-11.84)	7.4 (4-14.01)	<0.001
Cord insulin (mU/L) ‡	3.85 (2.3-6.5)	3.63 (2.2-6)	4.1 (2.41-6.92)	<0.001
SBP (mmHg) †	98.4 (11.1)	98.6 (10.9)	98.2 (11.2)	0.351
DBP (mmHg) †	61.6 (11.7)	60.9 (11.3)	62.2 (12.0)	0.007

*Difference between White British and Pakistani. †Mean (SD); ‡median (IQR); ‖n (%).

### Associations between biomarkers and adiposity measures

**Figure 1.**
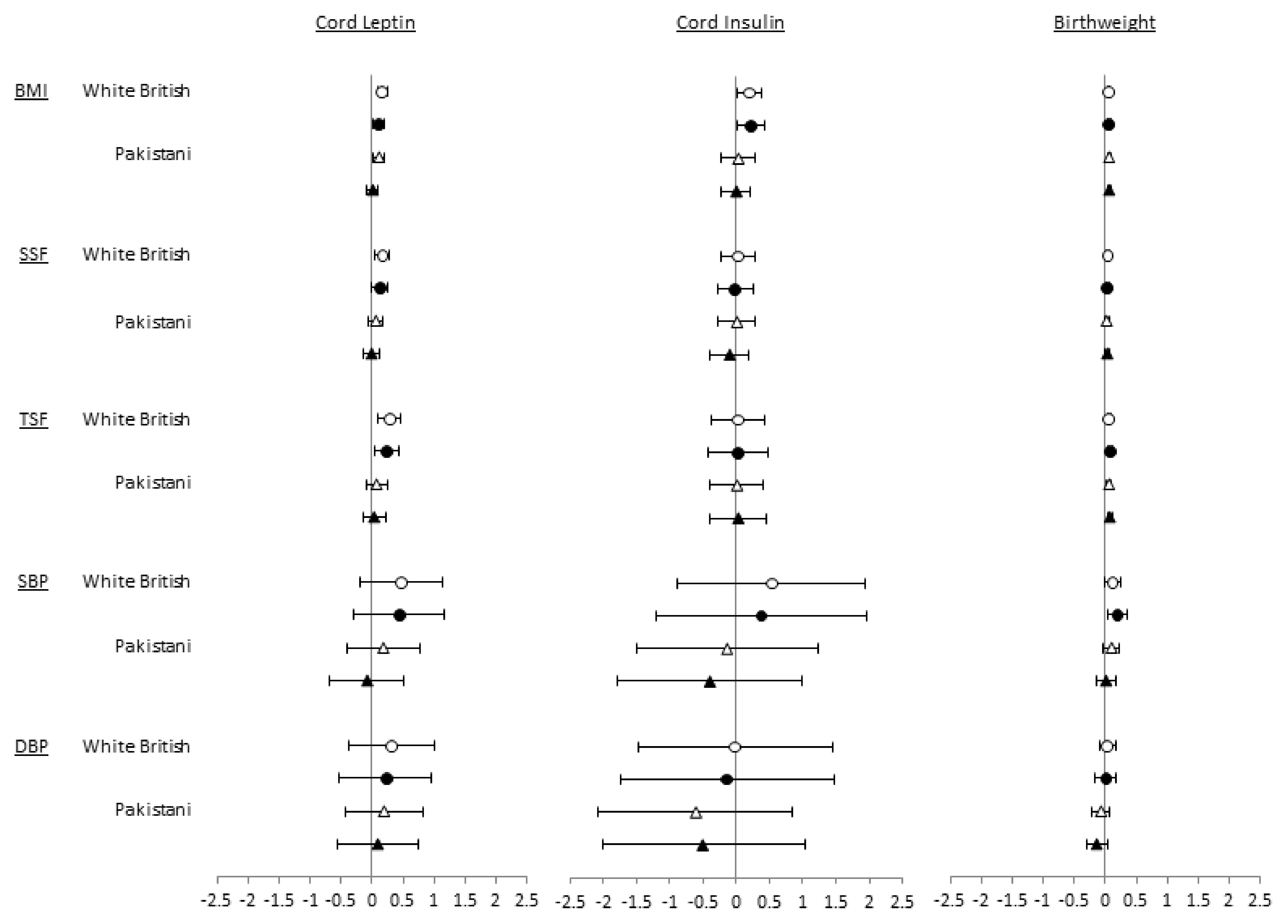
Associations of cord leptin, cord insulin and birthweight with adiposity and blood pressure in White British and Pakistani children at age 4/5 by ethnic group (models 1* and 2**). White circles: model 1*; black circles: model 2**. Values are differences in means (95% CI) of outcome per exposure unit or category. *Adjusted for offspring sex, age at measurement. **Adjusted for offspring sex, age at measurement, maternal age, smoking in pregnancy, parity, early pregnancy BMI, maternal education, gestational age, mode of delivery, HDP, GDM.

There were consistent positive associations of cord leptin with all adiposity outcomes in both ethnic groups in age- and sex-adjusted analyses (model 1) which attenuated towards the null with adjustment for all observed confounders in both ethnic groups (model 2) ( Figure 1 and *Extended data* Tables 1 and 2 ^20^). In the fully adjusted analyses (model 2), cord leptin was weakly positively associated with BMI, SSF and TSF in White British children with associations in Pakistani children somewhat closer to the null ( Figure 1 and *Extended data* Table 2 ^20^). However, results look very similar in each group and there was no statistical evidence that associations differed between the ethnic groups (all p-values for interaction terms ≥ 0.1). Cord insulin also appeared positively associated with BMI in White British children but not in Pakistani children, though again, results are very similar and there is no strong statistical evidence for a difference. Associations between cord insulin and TSF and SSF in White British and Pakistani children were all close to the null. Similarly, associations of cord leptin or insulin with SBP and DBP were close to the null and consistent in both ethnic groups. Birthweight had similar modest positive associations with all adiposity outcomes in both Pakistani and White British children; associations with SBP and DBP were close to the null in both groups.

**Figure 2.**
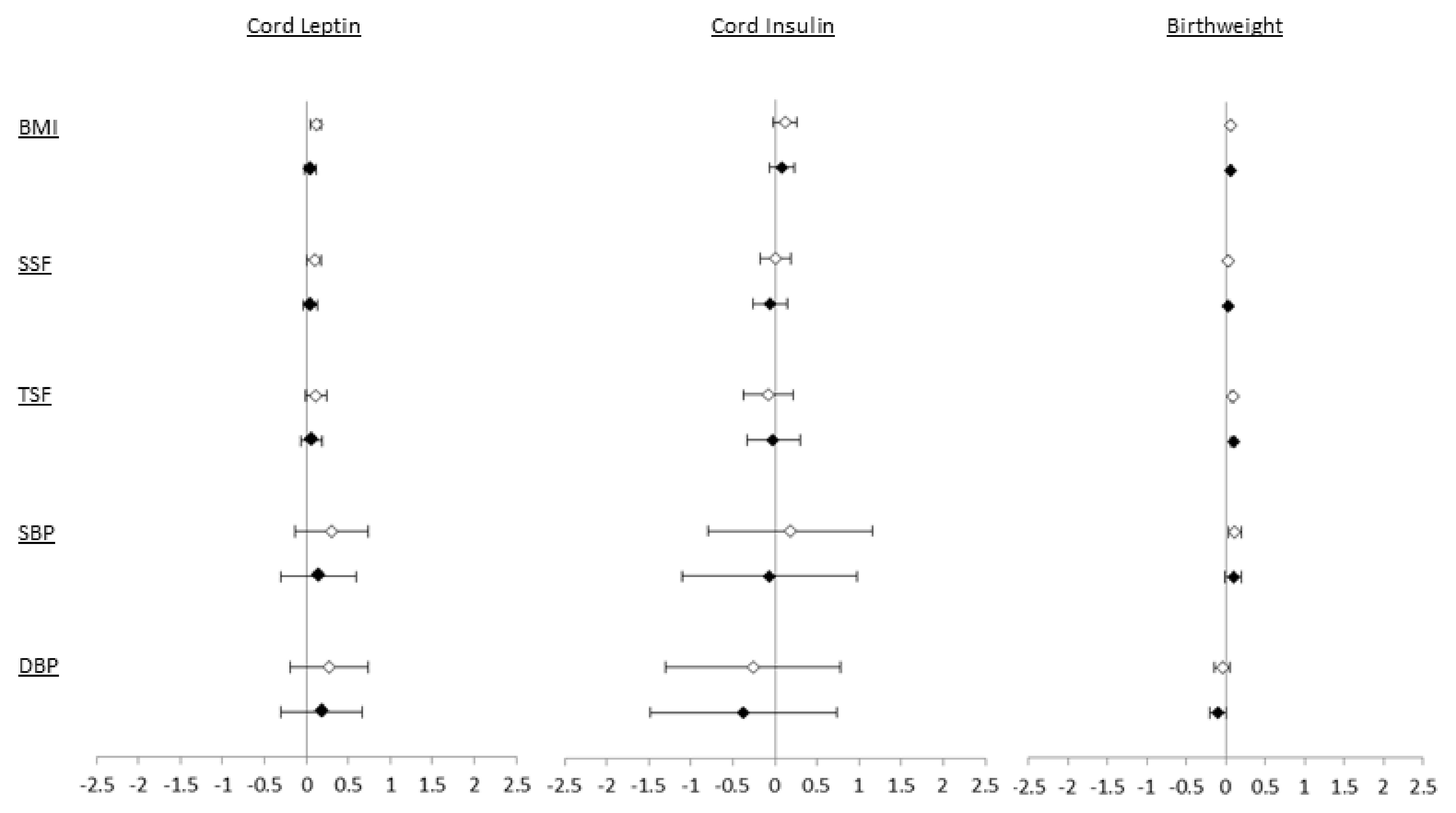
Associations of cord leptin, cord insulin and birthweight with adiposity and blood pressure in White British and Pakistani children combined (models 1* and 2**). White circles: model 1*; black circles: model 2**. Values are differences in means (95% CI) of outcome per exposure unit or category. *Adjusted for offspring sex, age at measurement. **Adjusted for offspring sex, age at measurement, maternal age, smoking in pregnancy, parity, early pregnancy BMI, maternal education, gestational age, mode of delivery, HDP, GDM. Notes: maternal age (years): continuous; smoking: smoking versus no smoking in pregnancy; parity: baseline 1; maternal BMI: difference in means per 1 kg/m2; education: baseline 5+ GCSEs; gestational age (weeks): continuous; mode of delivery: LSCS versus normal delivery; HDP: cases (either gestational hypertension or pre-eclampsia) versus non-cases GDM: cases versus non-cases.

Given the consistency of associations in Pakistani and White British children we present the confounder adjusted (model 2) results for both ethnic groups combined in Figure 2 and *Extended data* Table 3 ^20^. These show for associations of cord leptin and insulin with all outcomes point estimates are close to the null. Birthweight was weakly positively associated with measures of adiposity (BMI, SSF, TSF) but associations with SBP and DBP were close to the null.

## Discussion

Consistent with our previous findings ^5^, and using data from a far larger sample of the BiB cohort (2355 children), we have confirmed that cord leptin is higher in Pakistani compared to White British children, suggesting greater fatness at birth. Broadly consistent with other studies ^16, 18^ we have shown positive associations of birthweight with BMI, SSF and TSF in both White British and Pakistani children at age 4/5 years, but cord-blood leptin, a marker of fat mass ^14^ and insulin, a marker of fetal response to maternal gestational glucose, showed little evidence of important associations with anthropometric measurements. All of birthweight, cord leptin or cord insulin had little evidence of important associations with SBP and DBP at this age.

The modest associations of birthweight with all three of BMI, SSF and TSF in childhood in both ethnic groups are consistent with other studies (of largely White European populations) that show positive associations of birthweight with BMI, fat mass and lean mass ^18, 25^. Our findings for associations of cord leptin and insulin have some consistency with a previous study of mostly White Europeans, that reported a weak positive association of cord leptin with markers of adiposity at age 9–11, but not at age 17–18, and little evidence of cord-blood adiponectin with adiposity at age 9–11, but a weak positive association at 17–18 ^18^. Given the difference in body composition, greater risk of gestational and type 2 diabetes and of cardiovascular diseases in South Asian compared to White European populations ^1– 4^, we hypothesized that there may be differences in the relationships between cord leptin and insulin and childhood adiposity and blood pressure. However, we did not find evidence of ethnic differences and our results do not support an important association of cord leptin or insulin with early childhood adiposity or blood pressure.

Cord insulin may be a more direct reflection in the offspring, of adverse intrauterine exposures and is likely to reflect exposure to higher circulating maternal glucose. We have previously found no evidence that maternal gestational fasting glucose, post-load glucose or a diagnosis of GDM were importantly associated with later offspring adiposity or blood pressure or ethnic differences in these ^7, 8^. Given those earlier findings it is possibly unsurprising that we have found no association between cord insulin and adiposity or blood pressure in the same cohort. However, there may also be a genetic influence on fetal insulin secretion ^26^ and we had thought that cord leptin or insulin might provide useful biomarkers for risk prediction of future offspring adiposity and cardiovascular health, particularly in South Asian children. Given the lack of independent associations with outcomes in this cohort, it is unlikely that these are useful biomarkers.

The strengths of our study include a large sample size with cord blood samples and anthropometric measurements collected at birth and at age 4/5. Further, we have been able to compare ethnic differences in children who were all born in the same maternity hospital and are growing up in the same UK city, thus eliminating any potential variation by geography or health services. BiB has collected detailed information from participating families that has allowed important potential confounders to be controlled for. Although our sample size is large, we may lack statistical power to identify any ethnic differences, thus even larger studies may be required to further explore this further. That said our results did not suggest magnitudes of association that would likely be of public health importance. It has been suggested that SSF is a valid measure of centrally distributed fat and TSF of peripheral fat and these measures are available for a large number of BiB children. However, whilst skinfold measurements are feasible in large numbers, they may be less reliable than imaging techniques. We have included White British and Pakistani origin BiB children as these are the two main ethnic groups in the cohort. Some data are available for other ethnicities including other South Asian groups but the numbers available for analyses were too small for any meaningful analyses. As such our results may not be generalisable to other South Asian groups.

## Conclusion

Our results do not support the potential of either cord leptin or insulin to be useful biomarkers for the prediction of future offspring adiposity and cardiovascular risk in either White British or Pakistani children. Overall, we found no statistical differences between the ethnic groups for any of the associations tested. When both groups were combined, cord leptin and cord insulin were not found to be associated with markers of adiposity or BP at 4/5 years old. Further work is needed to better understand potential early markers of cardiovascular health in these two groups and any implications for prevention strategies or targeted interventions to prevent obesity and reduce cardiometabolic disease risk in later life and ethnic differences in these. For the time being, other factors such as family history and social-economic status may be more useful markers of child adiposity and cardiovascular risk.

## Data availability

### Underlying data

Scientists are encouraged and able to use BiB data, which are available through a system of managed open access. The steps below describe how to apply for access to BiB data.

- Before you contact BiB, please make sure you have read our Guidance for Collaborators. Our BiB executive review proposals on a monthly basis and we will endeavor to respond to your request as soon as possible. You can find out about the different datasets which are available here. If you are unsure if we have the data that you need please contact a member of the BiB team ( borninbradford@bthft.nhs.uk).
- Once you have formulated your request please complete the ‘Expression of Interest’ form available here and send to the BiB Programme Director ( rosie.mceachan@bthft.nhs.uk).
- If your request is approved we will ask you to sign a collaboration agreement and if your request involves biological samples we will ask you to complete a material transfer agreement.

### Extended data

Harvard Dataverse: Associations of cord leptin and cord insulin with adiposity and blood pressure in White British and Pakistani children aged 4/5. https://doi.org/10.7910/DVN/WDSB0J ^20^.

This project contains the following extended data:

- Extended data for Associations of cord leptin Aug 2019 (containing Extended data tables 1-3).
- Flowchart Leptin paper Aug 2019 (study flow chart).

### Reporting guidelines

Harvard Dataverse: STROBE checklist for ‘Associations of cord leptin and cord insulin with adiposity and blood pressure in White British and Pakistani children aged 4/5’ DRAFT VERSION.

https://doi.org/10.7910/DVN/WDSB0J ^20^.

Data are available under the terms of the Creative Commons Zero "No rights reserved" data waiver (CC0 1.0 Public domain dedication).

## References

[1] McKeigue PM Miller GJ Marmot MG: Coronary heart disease in south Asians overseas: a review. *J Clin Epidemiol.* 1989;42(7):597–609. 10.1016/0895-4356(89)90002-4 2668448

[2] McKeigue PM Ferrie JE Pierpoint T: Association of early-onset coronary heart disease in South Asian men with glucose intolerance and hyperinsulinemia. *Circulation.* 1993;87(1):152–161. 10.1161/01.cir.87.1.152 8419002

[3] Mather HM Chaturvedi N Fuller JH: Mortality and morbidity from diabetes in South Asians and Europeans: 11-year follow-up of the Southall Diabetes Survey, London, UK. *Diabet Med.* 1998;15(1):53–9. 10.1002/(SICI)1096-9136(199801)15:1<53::AID-DIA521>3.0.CO;2-V 9472864

[4] Ramachandran A Snehalatha C Vijay V: Low risk threshold for acquired diabetogenic factors in Asian Indians. *Diabetes Res Clin Pract.* 2004;65(3):189–195. 10.1016/j.diabres.2004.03.012 15331198

[5] West J Lawlor DA Fairley L: UK-born Pakistani-origin infants are relatively more adipose than white British infants: findings from 8704 mother-offspring pairs in the Born-in-Bradford prospective birth cohort. *J Epidemiol Community Health.* 2013;67(7):544–551. 10.1136/jech-2012-201891 23592862 PMC3859677

[6] West J Wright J Fairley L: Do ethnic differences in cord blood leptin levels differ by birthweight category? Findings from the Born in Bradford cohort study. *Int J Epidemiol.* 2013;43(1):249–254. 10.1093/ije/dyt225 24291804 PMC3937974

[7] West J Lawlor DA Santorelli G: Associations of social and economic and pregnancy exposures with blood pressure in UK White British and Pakistani children age 4/5. *Sci Rep.* 2018;8(1):8966. 10.1038/s41598-018-27316-1 29895845 PMC5997744

[8] West J Santorelli G Whincup PH: Association of maternal exposures with adiposity at age 4/5 years in white British and Pakistani children: findings from the Born in Bradford study. *Diabetologia.* 2018;61(1):242–252. 10.1007/s00125-017-4457-2 29064033 PMC6046463

[9] Tyrrell J Richmond RC Palmer TM: Genetic Evidence for Causal Relationships Between Maternal Obesity-Related Traits and Birth Weight. *JAMA.* 2016;315(11):1129–1140. 10.1001/jama.2016.1975 26978208 PMC4811305

[10] Aris IM Soh SE Tint MT: Associations of gestational glycemia and prepregnancy adiposity with offspring growth and adiposity in an Asian population. *Am J Clin Nutr.* 2015;102(5):1104–1112. 10.3945/ajcn.115.117614 26423388

[11] Ong KK Diderholm B Salzone G: Pregnancy insulin, glucose, and BMI contribute to birth outcomes in nondiabetic mothers. *Diabetes Care.* 2008;31(11):2193–2197. 10.2337/dc08-1111 18697902 PMC2571044

[12] Knight B Shields BM Hill A: The impact of maternal glycemia and obesity on early postnatal growth in a nondiabetic Caucasian population. *Diabetes Care.* 2007;30(4):777–783. 10.2337/dc06-1849 17251277

[13] Lawlor DA West J Fairley L: Pregnancy glycaemia and cord-blood levels of insulin and leptin in Pakistani and white British mother-offspring pairs: findings from a prospective pregnancy cohort. *Diabetologia.* 2014;5(12):2492–2500. 10.1007/s00125-014-3386-6 25273345 PMC4218974

[14] Hauguel-de Mouzon S Lepercq J Catalano P: The known and unknown of leptin in pregnancy. *Am J Obstet Gynecol.* 2006;194(6):1537–1545. 10.1016/j.ajog.2005.06.064 16731069

[15] Boeke CE Mantzoros CS Hughes MD: Differential associations of leptin with adiposity across early childhood. *Obesity (Silver Spring).* 2013;21(7):1430–1437. 10.1002/oby.20314 23408391 PMC3659179

[16] Mantzoros CS Rifas-Shiman SL Williams CJ: Cord blood leptin and adiponectin as predictors of adiposity in children at 3 years of age: a prospective cohort study. *Pediatrics.* 2009;123(2):682–689. 10.1542/peds.2008-0343 19171638 PMC2761663

[17] Lindsay RS Nelson SM Walker JD: Programming of adiposity in offspring of mothers with type 1 diabetes at age 7 years. *Diabetes Care.* 2010;33(5):1080–1085. 10.2337/dc09-1766 20427684 PMC2858180

[18] Simpson J Smith AD Fraser A: Programming of Adiposity in Childhood and Adolescence: Associations with birth weight and cord blood adipokines. *J Clin Endocrinol Metab.* 2016;102(2):499–506. 10.1210/jc.2016-2342 27841944 PMC5413167

[19] Wright J Small N Rayner P: Cohort profile: The Born in Bradford multi-ethnic family cohort study. *Int J Epidemiol.* 2013;42(4):978–91. 10.1093/ije/dys112 23064411

[20] West J: Associations of cord leptin and cord insulin with adiposity and blood pressure in White British and Pakistani children aged 4/5.2019; Harvard Dataverse, V1. 10.7910/DVN/WDSB0J PMC7475957 32954010

[21] World Health Organisation: Definition, diagnosis and classification of diabetes mellitus and its complications : report of a WHO consultation. Part 1, Diagnosis and classification of diabetes mellitus. 1999; accessed 28 July 2017. Reference Source

[22] West J Santorelli G Lennon L: Beyond height and weight: a programme of school nurse assessed skinfold measurements from white British and South Asian origin children aged 4-5 years within the Born in Bradford cohort study. *BMJ Open.* 2015;5(11):e008630. 10.1136/bmjopen-2015-008630 26610758 PMC4663422

[23] Lauer RM Clarke WR Beaglehole R: Level, trend, and variability of blood pressure during childhood: the Muscatine study. *Circulation.* 1984;6(2):242–249. 10.1161/01.cir.69.2.242 6690097

[24] Rao S Kanade A: Somatic disproportion predicts risk of high blood pressure among adolescent girls in India. *J Hypertens.* 2007;25(12):2383–2389. 10.1097/HJH.0b013e3282efff8e 17984658

[25] Brisbois TD Farmer AP McCargar LJ: Early markers of adult obesity: a review. *Obes Rev.* 2012;13(4):347–367. 10.1111/j.1467-789X.2011.00965.x 22171945 PMC3531624

[26] Shields BM Knight B Turner M: Paternal insulin resistance and its association with umbilical cord insulin concentrations. *Diabetologia.* 2006;49(11):2668–74. 10.1007/s00125-006-0282-8 16703330

