# Supplementary material for: Associations of cord leptin and cord insulin with adiposity and blood pressure in White British and Pakistani children aged 4/5 years
Source: Wellcome Open Res. 2019 Oct 15;4:157. [Version 1] doi: 10.12688/wellcomeopenres.15433.1 (PMC7475957; doi:10.12688/wellcomeopenres.15433.1)
Supplement: Peer review history

# Peer review: Reviewer response for version 1

**DOI:** 10.21956/wellcomeopenres.16879.r40104

**Published:** 2020-09-04

**Authors:** Harbindar Jeet Singh

**Affiliations:** 1 Department of Physiology, Faculty of Medicine, Universiti Teknologi MARA Sungai Buloh Campus, Selangor, Malaysia

In this study, the researchers aimed to ascertain whether cord blood leptin and insulin levels are associated with the level of adiposity in children at age 4 to 5 years. The rationale provided for this study was that the prevalence of total and central obesity is higher in adults of South Asian origin living in UK. As these are often associated with insulin resistance, risk of T2DM and cardiovascular disease, they wondered if leptin or insulin in cord blood could be used as early markers for these. My comments on the study are as follows.

 It is indeed a very challenging study to design and undertake, particularly when we know that the source of leptin in cord blood is both from the fetus and placenta. Their contributions to the cord blood leptin levels remain unknown. Any attempts at making correlations to outcomes later on in life would therefore be a little difficult. Besides this, there are also numerous factors that influence cord blood leptin levels. It is therefore not surprising that little or no significant association was detected between the two ethnic groups. Nevertheless, it is an interesting study that certainly adds useful information to the existing literature on this topic. 

 My other comments, which might be a little out of their aim of study, but nevertheless relevant, however include:

- It would have been good if they had also included data on placental weight, cord blood glucose concentration, maternal leptin levels, (although the latter may not influence the levels of leptin in cord blood very much), gestational age and ponderal index. Whilst these may not have altered their overall conclusion *per se*, these would have nevertheless aided in better interpretation of some of the data. 
- In the very early part of their discussion (page 6), the authors state the higher leptin levels in the cord blood suggest of greater fatness at birth in neonates of Pakistani origin. I am not too sure if this is convincingly supported by the data or that we can draw that conclusion without the benefit of other required parameters. To begin with, the mean birth weight (presumably also the % body fat) of neonates of Pakistani origin was lower than that of their white counterparts, and we would have therefore expected slightly lower cord blood leptin levels in these neonates of Pakistani origin. But that was not the case. Now, cord blood leptin concentration is known to correlate positively with birth weight, gestational age, birth height, ponderal index and even gender. In fact, higher levels of leptin in female neonates have been observed. From Table 1 it appears that there were more female infants in the Pakistani origin group than in the white British group. Could this have accounted for the higher cord blood leptin concentration in the Pakistani origin group? Smoking evidently suppresses leptin secretion, as cessation of smoking has been associated with increased circulating leptin levels in adults.There were more smokers in the white British group. How much, how long etc, etc are not stated. Could smoking during pregnancy influence leptin secretion, particularly from the placenta, if not from the fetus, and account for the lower leptin level in the cord blood from the white British group? These are some of the thoughts that come to mind immediately when perusing the data. Interestingly, there were more women with gestational hypertension and pre-eclampsia in the white British group. Leptin levels in serum and placenta are reportedly elevated in women with pre-eclampsia. Despite this the mean cord blood leptin level was still lower in the white British women than that in cord blood from women of Pakistani origin. It is also difficult to comment on the insulin levels as both leptin and glucose influence insulin levels. Clearly, given that a number of factors are known to influence cord blood leptin and insulin levels, these have to be taken into consideration when attempting to establish any association between cord blood leptin or insulin levels and adiposity or for that matter to any other condition in children or later on in adulthood.

Is the work clearly and accurately presented and does it cite the current literature?

Yes

If applicable, is the statistical analysis and its interpretation appropriate?

Yes

Are all the source data underlying the results available to ensure full reproducibility?

Yes

Is the study design appropriate and is the work technically sound?

Partly

Are the conclusions drawn adequately supported by the results?

Yes

Are sufficient details of methods and analysis provided to allow replication by others?

Yes

Reviewer Expertise:

Leptin in disease, hypertensive disorders of pregnancy, exercise physiology.

I confirm that I have read this submission and believe that I have an appropriate level of expertise to confirm that it is of an acceptable scientific standard.

# Peer review: Reviewer response for version 1

**DOI:** 10.21956/wellcomeopenres.16879.r37017

**Published:** 2019-11-29

**Authors:** Seeromanie Harding

**Affiliations:** 1 Department of Nutrition Science, School of Life Course Sciences, Faculty of Life Sciences & Medicine, King’s College London, London, UK

This paper ***“*** *Associations of cord leptin and cord insulin with adiposity and blood pressure in White British and Pakistani children aged 4/5 years* ***”*** is well written and helps to build the evidence base on the topic, which is sparse in relation to ethnicity. It shows positive associations of birthweight with adiposity indices in both White British and Pakistani children at age 4/5 years, but not with cord-blood leptin, a marker of fat mass and insulin. There was also no evidence for associations of cord leptin or cord insulin with blood pressure at this age.

 The study design is appropriate for the research question posed, and the statistical analyses and the conclusions drawn are adequately supported by the results. There are some minor issues that could be addressed to strengthen the reporting.

- On page 3, the authors of the study mention that *“Most studies have been small (<600 participants) and have reported positive and negative associations with childhood BMI.”* Given that only three studies have been published and reviewed by this study, it would be more accurate to say that few studies have been published on the topic, and these reported mixed results.
- On the same page (page 3), the authors mention that the analysis sample excluded children that attended a school outside the city of Bradford. What is the number of those children? How does attending a school in a neighbouring city affect the relationship between cord leptin or cord insulin with adiposity and cardiovascular outcomes in White British and Pakistani children? Were these children born and do they live in Bradford, but attended a primary school in a neighbouring city? Does the inclusion of these children change the reported results?
- On the issue of excluding 21.2% (n=635) of observations with missing data on the exposures, confounders and outcomes; Were those children with missing data systematically different from those children present in the complete case sample? The authors state that their analyses lack statistical power to identify any ethnic differences (page 7). If missing data are missing at random, multiple imputation could increase statistical power.
- Ethnic groups other than White British or Pakistani were excluded (n=858). It would be helpful to report the ethnic composition of this excluded sample. Growing diversity of cities in the UK is lived experience, such that in the future diversity could arguably be a more useful concept than conventional ethnicity labels. If included in the total – do the findings change? These issues could make a useful discussion point.
- In their conclusion, the authors highlight that *“For the time being, other factors such as family history and socio-economic status may be more useful markers of child adiposity and cardiovascular risk.”* However, no discussion is provided on the associations (and their magnitude) between control variables and the outcomes of interest. Maternal education was associated with both cord leptin/insulin and the outcomes of interest, but not with housing tenure and receipt of welfare benefits. It would be interesting to add some discussion around these findings. Was maternal education associated with housing tenure or welfare benefits? Would a better measure of socio-economic circumstances been a composite of all three indices to investigate associations with the extent of social adversity?
- Figure 2 - it is mentioned that birthweight is associated with all anthropometric outcomes (BMI, SSF and TSF). Please check as the point estimates for BMI and SSF appear to cross 0.

Is the work clearly and accurately presented and does it cite the current literature?

Yes

If applicable, is the statistical analysis and its interpretation appropriate?

Yes

Are all the source data underlying the results available to ensure full reproducibility?

Yes

Is the study design appropriate and is the work technically sound?

Yes

Are the conclusions drawn adequately supported by the results?

Yes

Are sufficient details of methods and analysis provided to allow replication by others?

Yes

Reviewer Expertise:

Social epidemiology.

I confirm that I have read this submission and believe that I have an appropriate level of expertise to confirm that it is of an acceptable scientific standard.
